# An exploratory study of men’s companionship, perceptions and experiences during pregnancy and delivery in Uganda

**DOI:** 10.1186/s12884-017-1385-6

**Published:** 2017-06-19

**Authors:** H. Lwanga, L. Atuyambe, H. Sempewo, A. Lumala, R. N. B. Byaruhanga

**Affiliations:** 1 St Francis Hospital Nsambya, Mother Kevin Post Graduate Medical School Uganda Martyrs University, Nkozi, Uganda; 2 Department of Community Health and Behavioural Sciences, Makerere University School of Public Health, College of Health Sciences, Kampala, Uganda

**Keywords:** Companionship during delivery, Men’s involvement, Uganda

## Abstract

**Background:**

Globally, low involvement of men in maternal health care services remains a problem to health care providers and policy makers. Men’s support is essential for making women’s world better. There are increasing debates among policymakers and researchers on the role of men in maternal health programs, which is a challenge in patriarchal societies like Uganda. The aim of the study was to assess companionship during delivery; men’s perception and experiences during pregnancy and delivery.

**Methods:**

This was a descriptive exploratory study using a qualitative approach. This study involved 16 male participants who were present in the labor room during the delivery of their child.

In-depth interviews (IDIs) were the main data collection methods used in the study. Purposive sampling was used to select participants who share particular characteristics with the potential of providing rich, relevant, and diverse data.

The interviews were tape-recorded with the permission of the participants; in addition, the interviewer took notes. Each interview lasted between 30 and 45 min. The transcripts were entered into ATLAS.ti for analysis. Manifest content analysis was used.

**Results:**

The major themes were; feelings about attending child birth, responsibilities during child birth, positive experiences and negative experiences about child birth. Men are willing to participate in child birth and should be encouraged as many are the decision makers in the family. Admission of men into the delivery room, improves family togetherness. The women felt loved and treasured. The men reported bondage to their partners and new born.

**Conclusions:**

Men’s involvement in the child birth process was associated with a more perceived bondage with the partner and the newborn. Their presence helped to promote a calm and successful child birth process. Hospitals should work on measures encouraging male involvement.

## Background

Men’s support is an essential component for making women’s world better. There are increasing debates among policymakers and researchers on the role of men in maternal health programs, which is a big challenge in Uganda where society is male driven.

The failure to incorporate men in maternal health promotion, prevention and care programs by policy makers, program planners and implementers of maternal health services has had a serious impact on the health of women, and the success of programs [1].

Traditionally, in other African settings, women have been attended and supported by other women during labour and birth [2]. Being decision makers, men’s involvement is likely to improve maternal and child health outcomes [3].

The tendency to view maternal health as a woman’s issue has contributed to a narrow focus of targeting mostly women. Social relationships determine people’s ability to manage their sexual and reproductive health (SRH) lives, with important implications not only to their health but also for other life choices [1].

According to the ICPD ten year progress report, a major remaining challenge is “the promotion of greater men’s responsibility in family and reproductive decision-making” on of greater men’s responsibility in family and reproductive decision-making” [4]. Active men’s involvement and participation in maternal health services is necessary to increase utilization of maternal health services by the pregnant women and mothers.

## Methods

These will deal with the research design, study population, the methods of data collection, processing, analysis and the limitations to the study.

An explorative qualitative design using in depth interviews was employed to explore the perceptions and experiences of male companionship during delivery [5] The study was carried out at a missionary private not for profit hospital providing quality care (St Francis Hospital Nsambya).

The study population comprised of all men of eighteen years and above who accompanied their spouses during labour and were willing to participate in the study. It is a missionary hospital located in the periurban area.

Its catchment population is approximately 250,000 with 7000 deliveries per year. Antenatal and maternity services at public health units in Uganda are provided free of charge. The Hospital, being a nongovernmental not- for- profit institution under Kampala Archdiocese, charges a fair user fee for these services.

Interviews were done for men who had accompanied their spouses during delivery until data saturation was reached. This was a point where no new information was gotten. Sixteen men were interviewed.

Purposive sampling was used to select participants who have characteristics with the potential of providing rich, relevant, and diverse data. The participants were approached in person, and none declined to participate.

Written consent was obtained from each of the men whose partner had normal vaginal delivery or caesarean section with a live birth in the hospital.

In depth interviews (IDIs) were the main data collection methods used in the study. Two (2) trained data collectors were used in the study. A pretested semi structured interview guide was used. I the principal researcher with the research assistant carried out the interviews. Interviews were conducted in English or Luganda, a local dialect in the periurban setting, depending on the language the respondent was comfortable with. Follow-up questions were asked and the clarification of points which arose was also sought.

One of the research assistants moderated and guided the interview while the other took notes and did the tape recording. The interviews were tape-recorded with the permission of the participants; in addition, the interviewer took notes. Codes were written on the interview guides and each recording was started by first mentioning the code on the interview guide to ensure data collected could be analyzed as belonging to that interviewee.

Notes were taken immediately after each interview. These notes covered the initial interviewee reactions, including the first analytical reflections from the interview content, and any useful observations that could not be captured by digital recording. Notes were taken on the demeanor of the respondent, his body language and mood, and any informal conversation that took place before or after the interview. Each interview ranged from 30 to 45 min.

The guide used covered the main areas on male companionship during labour. Questions were raised and participants were encouraged to express their opinion openly. Probes were made for clarification. Point of saturation was deemed to have been reached if no new information was obtained.

Validity in qualitative research is the extent to which observations reflect phenomenal of interest [6].

The principal researcher is a male doctor who conducted the interviews together with trained research assistance. The principal researcher was working at the Hospital while the research assistant in school of public health at a University. Data collection tools were pre tested and the semi structured interview guide was piloted to test the understanding of the questions.

Hence to improve credibility in data collection, clarification was sought during and after each interview. Triangulation was done to improve validity by interviewing women whose male partners participated in the study to collaborate their information.

Reliability in qualitative inquiry refers to the consistency of the research findings, through the interview process, the data transcription and analysis [6]. A semi structured interview guide was used to make sure that all participants answered the same questions. This improved consistency during interviews and build up of codes and themes. The data was transcribed and rechecked collaborating with the notes taken during interviews for gestures and motions, improving consistency.

Repeated listening to tapes of interviews was done however in absence of participants during analysis. Repeat interviews were done for 2 randomly selected participants to correlate with the initial interviews.

The taped interviews were transcribed verbatim after repeatedly listening to the recordings and the resulting texts analyzed by using Content *Analysis technique*. Broad themes were extracted from the transcripts and then progress to identifying coded themes. In establishing themes, consideration was given to statements of meaning that were present in most of the relevant data. In an attempt to ensure the credibility of the findings, an independent experienced coder was used to verify or corroborate the themes extracted from the data. The data was analyzed simultaneously with data collection. This allowed the researchers to progressively focus the interviews and observations, and to decide how to test the emerging conclusions. The transcripts were entered into ATLAS.ti for analysis. I developed a codebook based on the major themes of the study. The major themes were transformed in tree nodes and free nodes. Based on the codebook, I developed verified independently coded texts from the transcriptions.

The emergent themes and sub-themes are discussed below, supported and illuminated by respondents’ quotes. After exhaustive analysis of the interviews, it was possible to identify the following themes: feeling about attending child birth, responsibilities during child birth, positive experiences and negative experiences about child birth.

This research was approved by the Hospital’s Institutional Review Board (IRB) - IRC/PRJ/010/14/05. The data collection was carried out between October 2014 and January 2015, using semi-structured interviews, after being approved by the IRB of the fore mentioned institution and after the participants signed a Consent Form. To ensure confidentiality of participants who gave written consent, codes were used on the form instead of their names. In addition, only codes were written on interview transcripts.

The results cannot be generalized to all populations in and outside Uganda. We do not claim that these findings apply to all men who accompanied their partners during pregnancy and child birth.

The men who took part were married. Further exploration with men of different ethnicities, relationship status, and ages would be beneficial to expand on and compare with the findings in our study. The pre conception of the principal investigator from biomedical background training may affect the interpretation of information obtained from the interview.

## Results

### Demographic characteristics of the participants

All the participants were married and their ages ranged from 22 to 40 years with an average of 31 years (Table 1).

**Table 1:** Characteristics of the sample

Demographic characteristic	Number
Education	
Secondary	1
Tertiary	15
Tribe	
Ganda	8
Nyankole	2
Others	6
Parenthood	
First time fathers	6
Second and more time fathers	10
Spouses’ mode of birth	
Normal vaginal birth	12
Caesarean section	4

Four themes were chosen to describe the main findings of the study. They were constructed from several categories (Table 2).

**Table 2:** Themes and categories identified in interviews

Theme	Category
1. Feeling about attending child birth	Anxious and frightening
	Learning moment
	Great and comforting
2. Responsibilities during child birth	Supportive role
	Decision maker
3. Positive experiences during child birth	Planning family
	Joyful
	Bondage
4. Negative experiences during child birth	Painful
	Inability for men to be let in theatre
	unplanned reactions

The results showed that all the participants agreed about the importance of the presence of a companion during pregnancy and delivery, encouraging men’s involvement during delivery and have also recommended it to other men. This study involved 16 men who were present in the labor room during the delivery of their child. Their ages ranged from 22 to 40 years of age

Regardless of the education, parenthood, age and mode of delivery, the participants wanted men’s involvement increased during pregnancy and delivery. This made decision making easier, being supportive and a learning moment as well. In addition the majority of participants said attending child birth enabled them plan their family having seen what their partner goes through.

According to them, having been present during delivery has enhanced bondage to their child and spouse. Because, they participated in the pregnancy and delivery process, the participants have reached the conclusion that it causes physical pain on the part of the mother. In addition, they have claimed to feel the discomforts of delivery for example unplanned reactions and the uncertainty around the process.

### Feeling about attending child birth

#### Anxious and frightening

Nearly all participants reported anxiety and a feeling of being scared during childbirth until the moment the child is delivered and the feeling of happiness and joy. Before delivery the participants expressed that they did not know what was going to happen. The participants expressed that they should have been told what to expect during antenatal visits. First time fathers expressed anxiety and were scared that their partner did not know what to do during child birth process especially when it came to pushing the baby. A few respondents said the feeling was traumatizing. Nearly all participants reported that when any woman is delivering, it is a matter of life and death. There’s that feeling that something could go wrong. They always remain hopeful that nothing will go wrong.

*“To some extent it is frightening, you can see someone is in too much pain, to some extent you feel you are the cause of that pain, though you are not, though it is natural. Yah, but at the end of the day it is a tough experience.”* **(24 year old first time father)**

*“It’s a bit tense until the baby comes. That’s when I get relieved”* **(32 year old third time father)**

*“It is actually a scarily experience, very strange, for the first time I didn’t know what to expect, it is an encouraging one for the mother, at some point she could look at me and get the courage and I would encourage her to push.”* **(31 year old first time father)**

#### Great and comforting

Half the participants reported comforting their partners who told them the strength was gotten from their being around. Nearly all participants expressed that it is their obligation however some communication made them feel afraid and uncertain whether they will be going back with a baby or not. A few participants reported a better understanding of her partner and respecting her.

*“After the birth, I felt great. That was so good. Being even there, you know for the sex of the baby as well, I heard that actually the baby is a boy because the other ones have been girls so I was very happy about it”* **(38 year old fifth time father).**

#### Learning moment

Nearly all participants felt good at the opportunity of attending childbirth. A few participants reported tearing on seeing the baby. Participants reported that it was an emotional and learning moment. Participants felt responsible for the pain their partners were going through. They reported crying as they felt powerless and prayed to God for a good result.

*“Actually I felt better, I felt good, I felt very good and emotional, and I kept my tears until the day I saw her delivering the baby. The baby was getting out. It felt good; it actually showed me what women go through. I have gotten a better understanding of her; I have grown to respect her more. I respect her more; also I am still thinking whether I should have another child. Basically I have grown to respect her and respect every woman”* **(28 year old second time father)**

*“It was a learning curve I should say or a learning step for that matter. Many lessons to take from there which I don’t think we can exhaust in one day. So many things not to take for granted. The experience was so rich. It gives you what you can narrate to your wife after birth because basically she is in labour.”* **(30 year old first time father)**

### Responsibilities during child birth

#### Supportive role

Half the participants provided comfort to their partners during child birth. A few participants walked their partner around; many of them called them exercise. Nearly all participants played a supportive role by transporting their partners to hospital, picking things from the car, buying the ones that were urgently needed (financial support), massaging their partner, making tea and sometimes lifting. Participants caressed their partner’s back to reduce pain, holding her legs during delivery. A few participants reported to decrease pain and anxiety having a positive effect on the partner.

*“Actually I was so much giving comfort like holding her hands, giving support on the neck, also sometimes mainly translating, though she can understand, but being in pain, the language at times, she wasn’t doing what they could tell her, so I was telling her in my language to communicate I have been telling her to manage the pain by comforting.”* (**30 year old second time father)**

*“I had to make exercises with her which they ask them, it’s me who took her around the hospital. It’s me who was giving her hot water; it’s me who used to bring her for antenatal care. At least I made sure to remind her to take her medication according to prescriptions by the doctors during the pregnancy so I think I took full responsibilities of the pregnancy up to the date of birth”* **(29 year old second time father)**

*“My responsibilities were, the midwives were holding her legs and I was making sure that the head is just on her chest (demonstrates), this part was on her chest to make sure that the back doesn’t, she does not lift her bum, so I was making sure that the head was on the chest, to aid in the delivery.”* **(28 year old second time father)**

#### Decision makers

They were the decision makers especially when it came to going to theatre. A few of the participants reported that they were not prepared for theatre, and when it came to that time, they had to think about it. Their acceptance weighed on the explanation and communication made by the doctor. For participants whose partners were in pain were more likely to accept going to theatre than partners who weren’t. A few of participants reported that they decided when their partners would come for antenatal, when to come to hospital to deliver because it involved financial implications. Nearly all the respondents participated in choosing the hospital where partners would deliver from.

*“My responsibilities from the word go. I brought them to hospital. I was doing all the rounds. We need this, we need this. I was up and about. From 8am in the morning Sunday. I was supposed to consent to us making a decision to go for a C/section. I had to wait for her to come out of the theatre, bring her to her room, carry her off the stretcher to the bed.”* **(32 year old third time father)**

*“I would say I was like the main player. I had to do everything that was needed by that time. Yes I had sisters, I had other caretakers but I was the one who was supposed to be with her. Like massaging, massaging her, sometimes lifting her because she wanted that privacy, removing her clothes, putting them on, bathing her.”* **(27 year old second time father)**

*“It’s good to be around because you take a fast decision. Decision making becomes easy. You encourage the woman. The woman’s heart becomes strong and when she is pushing, she needs that, that (strength).* **(27 year old second time father)**

### Positive experiences during child birth

#### Planning family

Half the participants expressed that they will be planning their child birth having seen what their partners go through. One participant had planned for ten children but given the pain the partner had to bear to deliver their baby he changed his mind. Nearly all the participants reported child birth was a life and death experience. A few of participants reported understanding the role of a woman, if one didn’t know how someone comes to earth, it is really a lesson. It would really teach you a lot.

*“The experience really delivering is not easy. It’s really terrible. It’s about life and death. I have changed my mind now because I like children so much. I would like even to have ten but because of the experiences she is passing through I think four are enough. Four are enough for me, we will do whatever it takes at least to have the four girls are enough for me considering the experience yah the experience she is passing through that is over bleeding because I didn’t know that during delivery somebody can bleed like that”* **(40 year old seventh time father)**

#### Joyful

Nearly all participants reported feeling joyful and appreciated by their partners; one added his wife had never said good things from the bottom of her heart. Almost all the participants felt like men. Nearly all participants reported health workers being cooperative, friendly and had good customer care. Participants were so joyful to see the baby that they called everyone in their phone books.

*“Ok I danced. I laughed so there was joy all over me ok. And when am also joyous and when am happy, I talk a lot ok. So I made all the good comments. And I also appreciated my wife. Ok yah. There is another positive experience; my wife had never said whatever good thing I have done for her. She had never said from the bottom of her heart thank you for being there for me.”* **(36 year old third time father)**

Half the participants reported better bondage with their partners and new born. Participants reported family bondage being strengthened. A few of the respondents reported it as a partnership. If one has to be strong the other is encouraged and normally when a man is strong the woman becomes reassured.

*“To me it’s bondage of love because she feels like I was there when she needed me most”* **(27 year old second time father)**

### Negative experiences

#### Painful

Nearly all the participants reported child birth being a painful process. A few of the participants pointed out induced labour being more painful than normal one. Nearly all participants saw their partners in so much pain and felt powerless that they couldn’t do anything about it other than encourage their partners to be strong.

A few of the participants reported that as the pain increases they know the baby is coming. A few of participants reported the pain may be natural. Half the participants reported that by being around their partners were able to withstand the pain.

*“I think induced labour is so painful, because she had made ten months and they found it necessary to induce which I can’t justify, but the pain was too much. Even that’s what my wife is saying that even if not for herself she will tell others not to have induction, purely speaking”* **(30 year old second time father).**

*“The pain was too much for my wife to an extent that I dropped a tear I in that room.* “**(30 year old first time father)**

#### Inability for men to be let in theatre

Nearly all participants had a negative attitude towards delivery in theatre. A few of the participants reported not being ready to make the decision to go to theatre. They wanted their partner to experience normal delivery. They expressed their disappointment not being let into theatre to hear the cry of the baby. They were left outside theatre wondering what is going on. Theatre should have a provision for men to view what is going on to reduce anxiety.

*“It’s my wife who signed but I was called because I had stepped out so since it was an emergency, they called me and told me your wife is going to theatre so I said ok. When I reached, I went to theatre. It was annoying that I wasn’t allowed to theatre. It was also another by the way a negative experience that I wanted to go to enter because other theatres at least they allow you to peep from somewhere but here even mere opening the door to where they remove shoes, where they change from.”* (**36 year old first time father)**

#### Unplanned reactions

Half the participants reported bleeding being natural though they have fear as it is too much blood. A few participants reported being confused when something natural sometimes made the doctors panic. The number of doctors increases suddenly and you are left wondering what is happening developing pressure as well. Unplanned reactions made participants uncomfortable; many left the delivery room at that time to go stay with the baby.

*“It’s natural like during that it is a perception maybe but which can be dealt with because men are what they are so. You don’t know what will happen and it puts you somehow off. Sometimes you fear, sometimes you shake. You become confused and say now what is happening. Things are taking long. What is happening a doctor, even you start panicking doctor is everything ok. You start checking time. There is time even you can develop pressure because of that”* **(40 year old seventh time father)**

#### Factors affecting men’s involvement during labour

The factors that affect men’s involvement in maternal health care services may be categorized into Cultural factors, socio-economic, health facility factors, inter-spouse communication and perceptions men have on maternal health services.

Health facility factors included; Lack of privacy, comfort and lack of confidentiality, Poor attitude of health workers adversely affect men’s capacity to be involved in maternal health services.

Inter spouse communication; absence of inter-spousal communication and discussion can inhibit men’s involvement in maternal health care services. This prevents men from taking appropriate maternal health decisions.

Perceptions men have on maternal health services; perceptions that maternal health care services offered are poor and that services could be very expensive for both spouses to access.

## Discussion

Men’s involvement in the child birth process was associated with a more perceived bondage with the partner and the newborn. Their presence helped to promote a calm and successful child birth process. Four main themes were obtained: feelings about attending child birth, responsibilities during child birth, positive experiences and negative experiences about child birth. Men were willing to participate in child birth and should be encouraged as many are the decision makers in the family. Admission of a men into the delivery room, improves family togetherness. The women feel loved and treasured when a male companion was present during child birth.

This exploratory qualitative study provides an insight into the companionship during pregnancy and delivery, men’s experience during delivery in St, Francis Hospital Nsambya. The demographic data of the participants in this study showed that men who attended delivery were educated and profession men. These men could be likened to middle-class men in industrialized countries.

Regardless of the education, parenthood, age and mode of delivery, the participants wanted men’s involvement increased during pregnancy and delivery. This made decision making easier, being supportive and a learning moment as well. Although it is not a tradition for men to attend birth of their children, the participants demonstrated that men can be labour companions for their partners as women. Given the enabling environment, men can effectively assume the role of labour companions [7].

Men are willing to participate in labour and should be encouraged as many are decision makers in the family. This may have an impact on the three delays especially delay in seeking care, in recognising complications and asking for help and delay in reaching medical services. One male partner reported borrowing his neighbor’s car to bring his partner to hospital

The father in the labor room plays an essential role in the childbirth process by supporting and encouraging the mother to relax, which helps to promote a calm and successful labour and delivery process [8].

Male companions comfort their partners, massage their backs, prepare them tea, provide cotton and most of all, and learn how to take care of a pregnant mother in labor. This made decision making for emergency operation easier when the men were in the delivery room. The research shows hospital policies should advocate for men’s involvement especially provide space in theater where men can see the birth of their child, listen to the first cry as well.

Childbirth is the time when men are most receptive to getting involved with their families which makes men’s involvement critical for healthy pregnancy outcomes, infant survival and ideal child development [9].

The research has shown that the experience men go through during pregnancy and childbirth has a profound effect on planning their next child birth. Men expressed that they would not want their partner to go through child birth experience soon. One wanted 10 children but after seeing what his partner went through, he said he would stop at seven.

Admission of men into the delivery room, improves family togetherness, the women feel loved and treasured, the partners reported bondage to their partners and new borne. It was associated with high-level quality of intimate relationship between a man and a woman.

In agreement with prior research, the research demonstrated men’s attendance and active participation at labour and childbirth was associated with a belief of a ‘good’ partner, ‘good’ father [10] and a public statement of the strength of the father-infant bond [11].

The men stated that they were more knowledgeable about labour and appreciated the efforts of midwives and doctors in encouraging men’s attendance during labour. The men recognized the important role of partner’s presence played in promoting better care for their partners and new borne. One participant had to participate in translating for midwives what his wife was saying.

The participants expressed that they should have been told what to expect during antenatal visits. They expressed interest in attending antenatal with their partners to learn more about pregnancy and delivery. However due to commitments at their work places, majority are unable to attend antenatal visits but they made sure they attend delivery because they did not want second hand information about baby’s sex, time of delivery, condition of mother and baby. One participant cancelled a business trip to china to attend delivery of his child, an experience and decision he does not regret.

Participants reported that they decided when their partners would come for antenatal, when to come to hospital to deliver because it involved financial implications. The participants participated in choosing the hospital where partners would deliver from. This portrays the patriarch society where male companions during pregnancy and delivery are the decision makers.If male companionship is encouraged it could play a role in reduction in mortality and morbidity during pregnancy and delivery.

Male experience depended on various factors which included attitude of the health workers towards their partner during child birth, cost of services, preconceived traditional beliefs and lack of time off from their work places to attend child birth.

The factors that affect men’s involvement in maternal health care services may be categorized into Cultural factors, socio-economic, health facility factors, inter-spouse communication and perceptions men have on maternal health services.

Perceptions men have on maternal health services; perceptions that maternal health care services offered are poor and that services could be very expensive for both spouses to access [3].

Men expressed that they would have wanted to be present especially when main decisions were taken like being taken to theater.

## Conclusion

This will deal with the conclusion and recommendations

Men involvement in the child birth process was associated with a more perceived bondage with the partner and the newborn. Their presence helped to promote a calm and successful child birth process.

This study is very important for operational direction and improvement in service delivery. As a society, if men were more involved in pregnancy and child birth, they would bond more to their partners and newly borne, help to promote a calm and successful child birth and delivery process. However hospitals should work on measures encouraging men’s involvement, encouraging antenatal and delivery attendance. Provide a place in theatre where men can view as their babies are born. Their partners and health workers have shown to accept this approach.

### Recommendation

Men’s involvement during pregnancy and delivery should be encouraged as men are willing to participate, improves family bondage, makes delivery process bearable for the spouse and decision making quicker. Further research is needed to find out if women would want their partners to be let in theater during delivery of the child.

Men may need education about labour and childbirth processes so that they are aware of what to expect when they accompany their partners for labour. This awareness may enable them to support their partners emotionally throughout labour.

Further exploration with different ethnicities, relationship status, and ages would be beneficial to expand on and compare with the findings in our study.

## Abbreviations

ANC: Antenatal care
FGD: Focus group discussion
FP: Family planning
HSSP: Health sector strategic plan
ICPD: International conference on population and development
IEC: Information education and communication
MCH: Maternal and child health
MDGs: Millennium development goals
MOH: Ministry of health
PPASA: Planned Parenthood Association
SRH: Sexual and reproductive health
UDHS: Uganda demographic health survey
UNFPA: United Nations Population Fund
UNICEF: United Nations Children’s Fund
WHA: World Health Assembly
WHO: World Health Organization

## References

[1] Greene M, Puherwitz MM, Deirdre W, Akinrinola B, Susheela S (2003). Involving men in reproductive health.

[2] Kululanga LI, Sundby J, Malata A, Chirwa E (2011). Striving to promote male involvement in maternal health care in rural and urban settings in Malawi - a qualitative study. Reprod Health.

[3] Nantamu P (2011). Factors associated with male involvement in maternal health care services in Jinja District, Uganda.

[4] UNFPA (2004). In people: national progress in implementing the ICPD program of action 1994–2004.

[5] Tucker M (2000). Qualitative research methods.

[6] Kvale S. Interviews: an introduction to qualitative research interviewing. Thousands oak: Sage publications; 1996.

[7] Kululanga LI, Malata A, Chirwa E, Sundby J (2012). Malawian fathers’ views and experiences of attending the birth of their children: a qualitative study. BMC Pregnancy Childbirth.

[8] Maria R (2011). The fathers’ perception about their presence in the labor room during the birth of their child: a descriptive study.

[9] Kaplan W (2004). New dads in labor: an opportunity for involvement. Int J Childbirth Ed.

[10] Wertz R (2001). lying-in: a history of childbirth in America. Int J Consum Stud.

[11] Early R (2001). Men as consumers of maternity services: a contradiction in terms. Int J Consum Stud.

